# Tissue specific age acceleration patterns in the sperm of oligozoospermic men

**DOI:** 10.3389/frph.2022.1043904

**Published:** 2022-11-23

**Authors:** Kelaney Stalker, Chad Pollard, Kenneth Aston, Tim Jenkins

**Affiliations:** ^1^ Department of Cell Biology and Physiology, Brigham Young University, Provo, UT, United States; ^2^ Department of Surgery, Division of Urology, University of Utah, Salt Lake City, UT, United States

**Keywords:** age calculator, epigenetics, oligozoospermia, DNA methylation, sperm tissue specific aging

## Abstract

To determine if disease can modify aging patterns in an affected tissue without altering the aging patterns of other tissues, blood and semen of individuals with oligozoospermia (*n* = 10) were compared to the blood and semen of individuals with normozoospermia (*n* = 24). DNA methylation data was obtained *via* Illumina's 850 K array. The Horvath and Jenkins age calculators were then utilized to predict the epigenetic age of blood and sperm. Epigenetic age of sperm was approximated using germ-line age differential (GLAD) values. Using nonpaired *t*-tests, it was found that sperm of oligozoospermic men (mean GLAD score of 0.078) were predicted to be significantly older than the sperm of normozoospermic men (mean GLAD score of −0.017), returning a *p*-value of 0.03. However, there was not a significant epigenetic age difference between the blood of those with oligozoospermia (mean GLAD equivalent score of −0.027) and normozoospermia (mean GLAD equivalent score of 0.048), producing a *p*-value of 0.20. These results lead to the conclusion that tissue specific aging is occurring in sperm of oligozoospermic individuals but not in unaffected somatic tissues (in this case, blood).

## Introduction

Aging is an inevitable part of life. Perhaps because of its invariance, senescence has long fascinated scientists in various fields. This interest in understanding the etiology of aging has led to many discoveries, such as various genetic and epigenetic marks changing consistently with age. This has been true in the assessment of telomere length and, more recently, in studies of DNA methylation. These measures of cellular aging have been utilized to generate age calculators. The utilization of DNA methylation to predict an individual's age has been particularly effective. In 2013, Steven Horvath produced the first DNA methylation age calculator. This calculation has proven to be effective in many different somatic tissues in humans. While it is most commonly used in blood, it has been proven to effectively predict age based on methylation in many tissue types; however, it proved ineffective at accurately predicting chronological age in sperm (1). Based in part on data produced in 2014 in sperm, the Jenkins lab produced a sperm DNA methylation age calculator that was able to predict age in sperm with similar accuracy to the Horvath calculator in somatic tissues (2). This finding has allowed the accurate assessment of epigenetic age prediction in both somatic tissue and the germ line. Both calculators are publicly available online (https://dnamage.genetics.ucla.edu/home; https://github.com/timgjenkins/Jenkins-et-al-2017).

Sperm DNA methylation patterns can be altered by environmental factors such as exposure to toxins, cigarette smoke, and various diseases and states of infertility (3). This shift in sperm methylation patterns can also be reflected in predicted epigenetic age. In fact, it has been hypothesized that epigenetic aging may be a better indicator of biological age than an individual's chronological age alone, as it likely takes into account the cumulative impact of environmental exposures accrued over an individual's life. However, it remains unclear if epigenetic age can be tissue specific and not uniformly altered within an entire individual. This is particularly interesting where a disease state is found in a single tissue, while the rest of individual's tissues appear to be healthy. One such example is found in the case of oligozoospermia where there is a significant deficit in sperm production, but often (though not always) these individuals are otherwise healthy. Herein, we aim to explore if disease state can affect the pattern of aging in tissues impacted by disease and can cause the affected tissue to age independently of other tissues in the body. To do so, blood and semen from individuals diagnosed with normozoospermia or oligozoospermia were compared.

## Methods and materials

**Figure 1:**
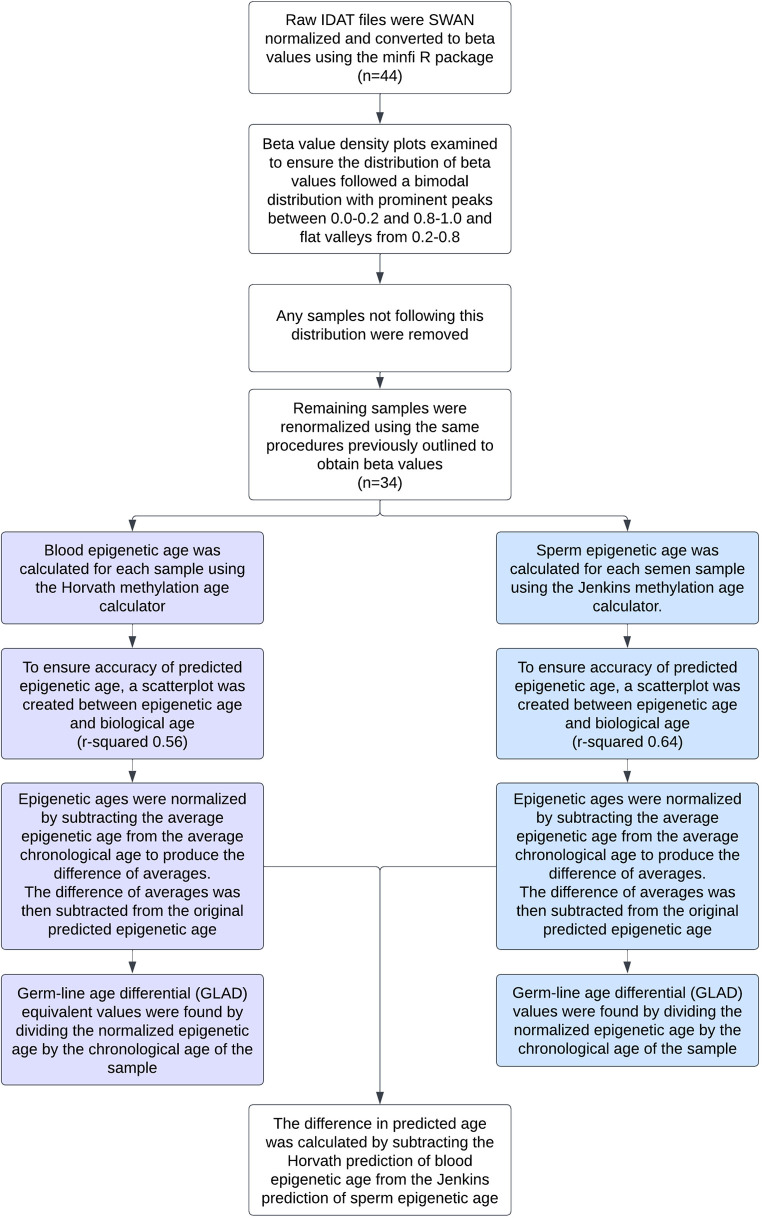
Flow chart detailing the data preprocessing and statistical analyses performed on each sample.

A flow chart depicting all methods used can be seen in Figure 1.

### Sample preparation

To isolate DNA, sperm samples were thawed simultaneously and were subjected to a column-based DNA extraction protocol with sperm-specific modification to the DNeasy kit (Qiagen). To eliminate white blood cell contamination, prior to DNA extraction, somatic cell lysis was performed by incubation in somatic cell lysis buffer (0.1% sodium dodecyl sulfate, 0.5% Triton X-100 in diethylpyrocarbonate H2O) for 20 min on ice. After somatic cell lysis, a visual inspection of each sample was performed to ensure the absence of all potentially contaminating cells before proceeding.

Extracted sperm DNA was bisulfite converted with the EZ-96 DNA Methylation-Gold kit (Zymo Research) according to manufacturer recommendations specifically for use with array platforms. The converted DNA was delivered to the University of Utah Genomics Core Facility and hybridized to Infinium HumanMethylation450 BeadChip microarrays (Illumina) and analyzed according to manufacturer protocols. All sample preparation was performed as described by Aston, et al. (4).

### Data preprocessing

Raw IDAT methylation array data from all samples was preprocessed using the minfi R package. Data was SWAN normalized to produce beta values for each cytosine-guanine dinucleotide (CpG). Density plots of the beta value distribution of each sample were examined to ensure the distribution of beta values was bimodal in nature with prominent peaks between 0.0–0.2 and 0.8–1.0 and a flat valley from 0.2–0.8. Any samples not following this distribution were removed. These qualitative methods were also confirmed by using minfi to calculate median intensity scores. All samples that had a median intensity score below the standard were removed. The remaining samples were renormalized using the same procedures previously outlined. To verify the absence of somatic cell contamination, methylation at DLK1 was assessed (2). The mean beta value of each sample at DLK1 was calculated and fell within the accepted threshold of ∼0.25 (Supplementary Figure S1). To ensure age was not a confounding variable, the standard error of the chronological age of the oligozoospermic cohort (32.73 ± 1.9) and normozoospermic cohort (31.64 ± 1.3) were calculated and a heteroscedastic t-test performed (*p*-value of 0.61).

### Statistical analysis

**Figure 2:**
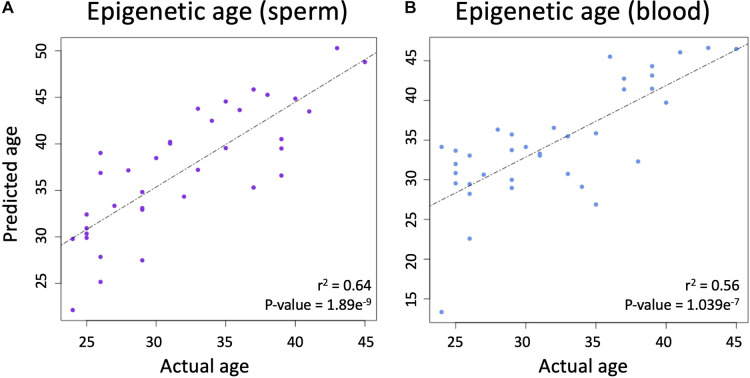
(**A**) The relationship between chronological age and epigenetic predicted age in sperm. (**B**) The relationship between chronological age and epigenetic predicted age in blood.

Using the produced beta values, epigenetic ages were calculated for both blood and sperm. Blood epigenetic age was calculated for each somatic sample using the Horvath methylation age calculator. Sperm epigenetic age was calculated for each semen sample using the Jenkins methylation age calculator. Steps on how to run each calculator are included on the corresponding websites (https://dnamage.genetics.ucla.edu/home; https://github.com/timgjenkins/Jenkins-et-al-2017). To ensure accuracy of predicted epigenetic age, scatterplots were created (Figure 2).

The resulting epigenetic ages were then normalized by subtracting the average epigenetic age from the average chronological age to produce the difference of averages. This difference was then subtracted from the original predicted epigenetic ages to produce adjusted epigenetic ages with matched means to the original ages. Germ-line age differential (GLAD) values were found by dividing the normalized epigenetic age by the chronological age of the sample to help avoid issues of heteroskedasticity that can be found when subtracting the actual and predicted ages. The difference in predicted age was calculated by subtracting the Horvath prediction of blood epigenetic age from the Jenkins prediction of sperm epigenetic age. The analysis can be replicated following the instructions found at https://github.com/jenkins-lab-byu/TSA_Project).

## Results

### Linear regression

A linear regression analysis between the predicted and actual ages in sperm produced an adjusted r-squared statistic of 0.64 and a *p*-value of 1.869 × 10^−9^ (Figure 2A), while Horvath's blood epigenetic calculator produced an adjusted r-squared statistic of 0.56 and a *p*-value of 1.039 × 10^−7^ (Figure 2B) while using the same statistical tools. These statistics allow for the conclusion that epigenetic age predictions were accurate in both blood and sperm.

### T-tests

**Figure 3:**
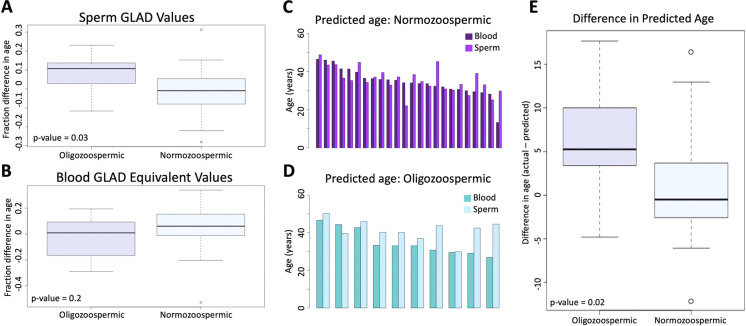
(**A**) the difference between oligozoospermic and normozoospermic germline age differential values. (**B**) The equivalent relationship as 3A is demonstrated in blood. (**C**) The difference in predicted age of blood and sperm of normozoospermic individuals. (**D**) The difference in predicted age of blood and sperm of oligozoospermic individuals. (**E**) The mean difference of the predicted epigenetic age of sperm in oligozoospermic and normozoospermic individuals.

A pairwise *t*-test was used to assess if adjusted GLAD values were significantly different between oligozoospermic samples (average in sperm = 0.078; average in blood = −0.027) and normozoospermic samples (average in sperm = −0.017; average in blood = 0.048). In sperm, the test returned a *p*-value of 0.03 (Figure 3A), but in blood, the *p*-value was 0.2 (Figure 3B). Difference in predicted epigenetic age in both blood and sperm is illustrated in Figures 3C,D. A *t*-test assessing the difference of predicted age between oligozoospermic and normozoospermic samples produced a *p*-value of 0.02 (Figure 3E). Sperm of those with oligozoospermia were predicted to be significantly older than chronological age, but in blood, epigenetic age prediction was not significantly different from chronological age. Normozoospermic blood and sperm epigenetic predictions matched chronological age (*p*-value of 0.61).

## Discussion

We set out to explore the potential for tissue specific aging patterns in cells (sperm) that we know are directly impacted by a disease state while also assessing a paired tissue (blood) from the same individual that appear to be unaffected by the disease state. Our data suggest that oligozoospermic individuals have significantly accelerated epigenetic aging profiles in their sperm compared to normozoospermic individuals. Importantly, this age acceleration pattern is tissue specific and seen only in the affected tissue and not in blood. This increase in age in sperm is illustrated by a GLAD score, which acts as a broad indicator of epigenetic health and provides the percent alteration in epigenetic aging in a given tissue. An increased GLAD score represents sperm with an older (or accelerated) epigenetic signature than their chronological age. These patterns of accelerated aging are particularly intriguing when taking into account that previous research has shown that the progeny of older fathers have an increased incidence of various neuropsychiatric disorders and trinucleotide expansion associated diseases (5). To be clear, there are no data that implicate the increased aging detected using epigenetic aging calculations in an elevated risk of age associated outcomes in the offspring, but findings such as those presented in this study should be taken into consideration as we learn more about aging in the gamete and the downstream implications of an “aged” epigenetic profile.

Because epigenetic signatures are unique to cell type, tissues selected for future analyses of aging should be carefully purified to ensure accurate epigenetic age prediction (6). It will be important to consider that, based on these data, a disease state could potentially act as a confounding variable. Thus, unexpected increases in epigenetic age could be due solely to disease state, which may be problematic for some future studies.

That accelerated aging is only reflected in sperm and not blood in our study suggests a tissue specific aging pattern. This is a novel finding in the study of male infertility, but also in the wider field of epigenetics as the implications of tissue specific epigenetic aging may be of consequence in many different disease states and tissues. Because this work represents a pilot study it should be replicated in a larger cohort with appropriate meta data to control for other factors known to affect sperm methylation patterns, such as smoking, obesity, diet, and ethnicity. Further exploration into this topic is needed broadly, but also specifically in the case of male infertility. Future studies should focus on the impact of different disease states and their impact on epigenetic age. Additional research in the fertility space needs to determine if this trend is true for other fertility abnormalities in men (teratozoospermia, asthenozoospermia, etc.), determine the impact of epigenetic aging directly on pregnancy outcomes, and to determine if any intervention can rescue accelerated aging patterns.

## Data Availability

The datasets presented in this study can be found in online repositories. The names of the repository/repositories and accession number(s) can be found in the article/**Supplementary Material**.
