# Supplementary figures and images for: Tissue specific age acceleration patterns in the sperm of oligozoospermic men
Source: Front Reprod Health. 2022 Nov 23;4:1043904. doi: 10.3389/frph.2022.1043904 (PMC9727134; doi:10.3389/frph.2022.1043904)

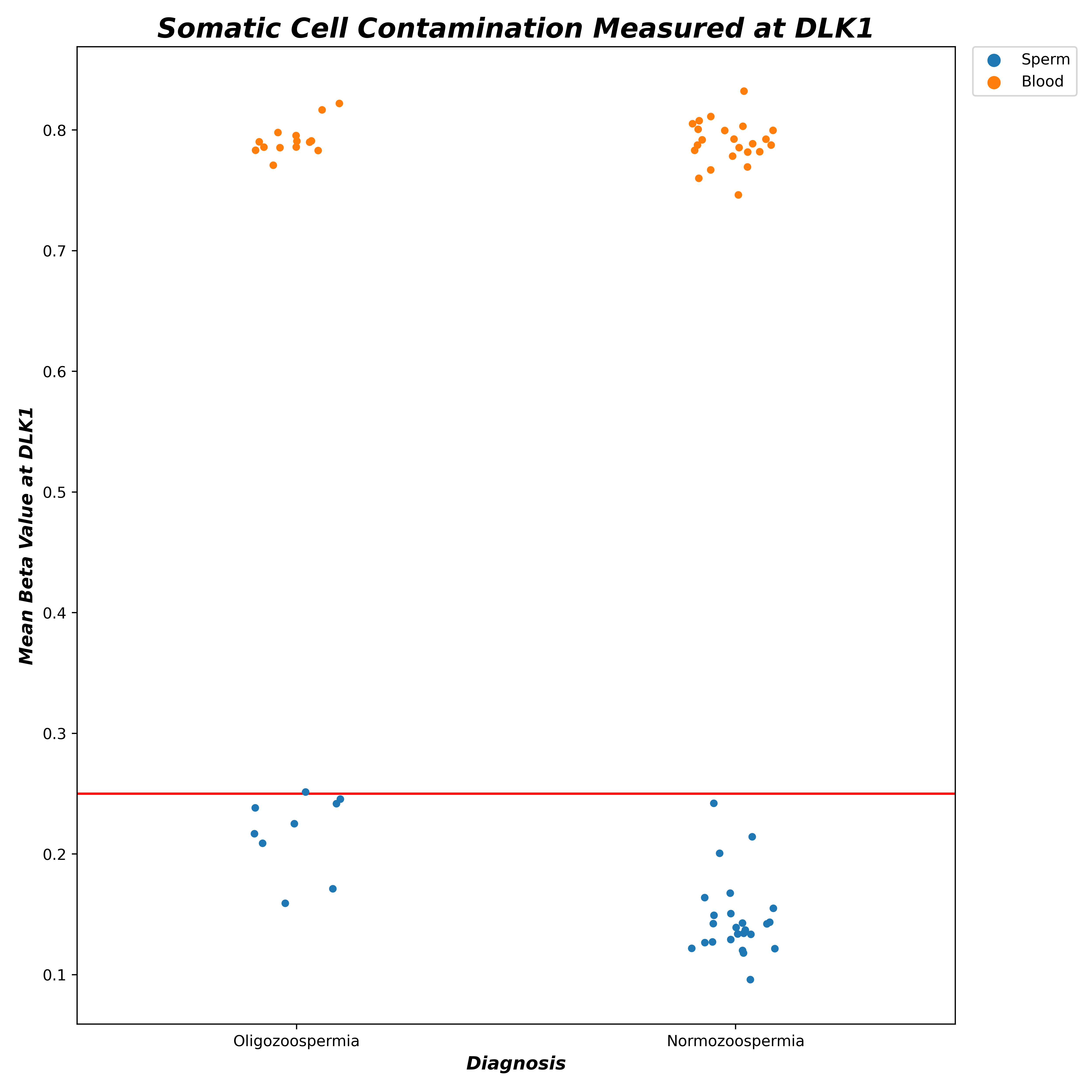

Supplement: Supplementary file 1 [file Image1.jpeg]
